# Role of National Travel Health Network and Centre Website during Pandemic (H1N1) 2009

**DOI:** 10.3201/eid1701.100486

**Published:** 2011-01

**Authors:** Nicola Boddington, Naomi Bryant, David R. Hill

**Affiliations:** Author affiliations: National Travel Health Network and Centre, London UK (N. Boddington, N. Bryant, D.R. Hill);; London School of Hygiene and Tropical Medicine, London (D.R. Hill)

**Keywords:** Influenza, pandemic, website, information systems, Internet, travel medicine, viruses, Mexico, letter

**To the Editor:** The National Travel Health Network and Centre (NaTHNaC) was created in 2002 by the Department of Health in England to provide authoritative guidance in travel medicine. The open-access NaTHNaC website (www.nathnac.org) is a key mode of communication, with both health professionals’ and travelers’ areas. Website country information pages (CIP) provide specific guidance for travel to each country of the world, and an outbreak surveillance database (OSD) detailing global outbreaks of disease is updated daily.

In late April 2009, influenza A virus (H1N1) of swine origin was identified in 2 children from California, USA (*1*). These cases were traced to travel to Mexico, and a widespread outbreak of influenza A (H1N1) in Mexico subsequently was recognized. On June 11, 2009, the **World Health Organization declared a global influenza pandemic (*****2*****). We reviewed use of the** NaTHNaC website during the early recognition of pandemic (H1N1) 2009. During this phase, before widespread community transmission in the United Kingdom, assessing the international situation was necessary because travel abroad represented the highest risk for infection (*3*).

NaTHNaC, the national authority for travel health advice, posted multiple information resources on pandemic (H1N1) 2009. A daily table of internationally reported cases and deaths was compiled from official sources. A more detailed report of confirmed and suspected cases was circulated to key NaTHNaC stakeholders, including the Health Protection Agency (HPA) and the Foreign and Commonwealth Office (FCO). The OSD listed progression of the pandemic by date, country, and region. Reports of the pandemic and advice on preventive measures for travelers, termed Clinical Updates, were written daily, posted, and circulated to stakeholders.

NaTHNaC website statistics were obtained from Google Analytics. Use for the first 8 weeks of the pandemic period (April 24–June 18, 2009) was extracted, analyzed by using STATA version 9.1 (StataCorp LP, College Station, TX, USA), and compared with use for the 8 weeks preceding the start of the pandemic influenza (prepandemic period, February 27–April 23, 2009).

During the pandemic period, the daily number of visits to the website increased 28.1% over the prepandemic period (Table; Technical Appendix Figure 1). More new visitors accessed the website (63.6% vs. 61.7%), particularly through the Health Professionals portal (50.7% vs. 46.1%; p<0.001).

**Table:** Number of visits to or searches on the National Travel Health Network and Centre website during the 8 weeks before and after recognition of pandemic (H1N1) 2009

Visits or searches	No. before*	No. after†	% Change
Daily website visits	1,664 ± 655	2,132 ± 885	+28.1
Website access from Mexico	55	210	+281.8
Visits to Mexico Country Information Page	2,040	4,090	+100.5
Mexico searches on the Outbreak Surveillance Database	50	459	+818.0
Visits to US country information page	654	2003	+206.3
Visits to seasonal influenza information sheet	34	1,572	+4,523.5
Visits to Outbreak Surveillance Database home page	2,050	5,110	+149.3
Referral traffic from Foreign and Commonwealth Office	21,604	31,200	+44.4
Referral traffic from Health Protection Agency	1,399	7,247	+418.0

*Data from before the beginning of the pandemic (February 27–April 23, 2009). †Data from the first 8 weeks of the pandemic (April 24–June 18, 2009).

The number of website visitors from Mexico and the number of visits to the Mexico CIP also increased; Mexico was the most frequently searched country on the OSD (Table). Visits to the Mexico CIP (633 visits) and the Mexico OSD (129 visits) pages peaked on April 27, the Monday after pandemic (H1N1) 2009 was recognized. The pandemic (H1N1) 2009 home page that hosted clinical updates, news items, and an information sheet about subtype H1N1 became the seventh most viewed page (11,009 views). Visits for advice on seasonal influenza also increased markedly.

During the pandemic period, the website was accessed more often through referring websites (46.3%) than it was during the prepandemic period (39.9%; p<0.001). The most frequent referral website was the FCO (Table), accounting for 56.4% of all referrals during the pandemic period, with a peak on April 27 (Technical Appendix Figure 2). A large increase also occurred in referrals from the HPA.

Our analysis documents increased use of a national resource during the emergence of pandemic (H1N1) 2009. Information accessed included specific country information for Mexico and the United States, the countries first reporting cases, and information about and guidance for the prevention of pandemic (H1N1) 2009. The 28% increase in access to the website most likely reflected widespread interest in the pandemic, new links to the NaTHNaC website from UK authorities (e.g., FCO and HPA), and daily communication with stakeholders within the United Kingdom. In addition, NaTHNaC collaborated with these stakeholders and public health agencies to report progression of the outbreak and to help set policy on travel to influenza-affected countries.

The internet is a major resource for travel health information for health professionals and travelers. In 2008, ≈83% of internet users and 61% of all US adults used the Internet to acquire health information; 9% searched for travel health information (*4*). Public health agencies also use the Internet to assess global disease threats. Many use informal Internet sources, such as news articles and media outlets, to monitor potential threats in a more timely fashion than through the often delayed public health reporting mechanisms (*5**–**7*).

During a rapidly evolving global health situation, such as pandemic influenza, timely, accurate information is needed. The World Health Organization provided daily, and often twice daily, information (*8*); the US Centers for Disease Control and Prevention and the European Centre for Disease Prevention and Control used new and existing reporting systems (*9**,**10*). The experience of NaTHNaC indicates that acquisition and coordination of information with health authorities, rapid and direct communication of findings and recommendations to stakeholders, and posting of this information for access by travelers and health professionals can increase communication about global health events.

## Supplementary Material

Appendix Includes Technical Appendix Figure 1 and Technical Appendix Figure 2.
