# Supplementary material for: Role of National Travel Health Network and Centre Website during Pandemic (H1N1) 2009
Source: Emerg Infect Dis. 2011 Jan;17(1):149–50. doi: 10.3201/eid1701.100486 (PMC3204626; doi:10.3201/eid1701.100486)
Supplement: Appendix — Includes Technical Appendix Figure 1 and Technical Appendix Figure 2. [file 10-0486-Techapp.pdf]

Article DOI: 10.3201/eid1701.100486

Role of National Travel Health Network and Centre Website during Pandemic (H1N1) 2009

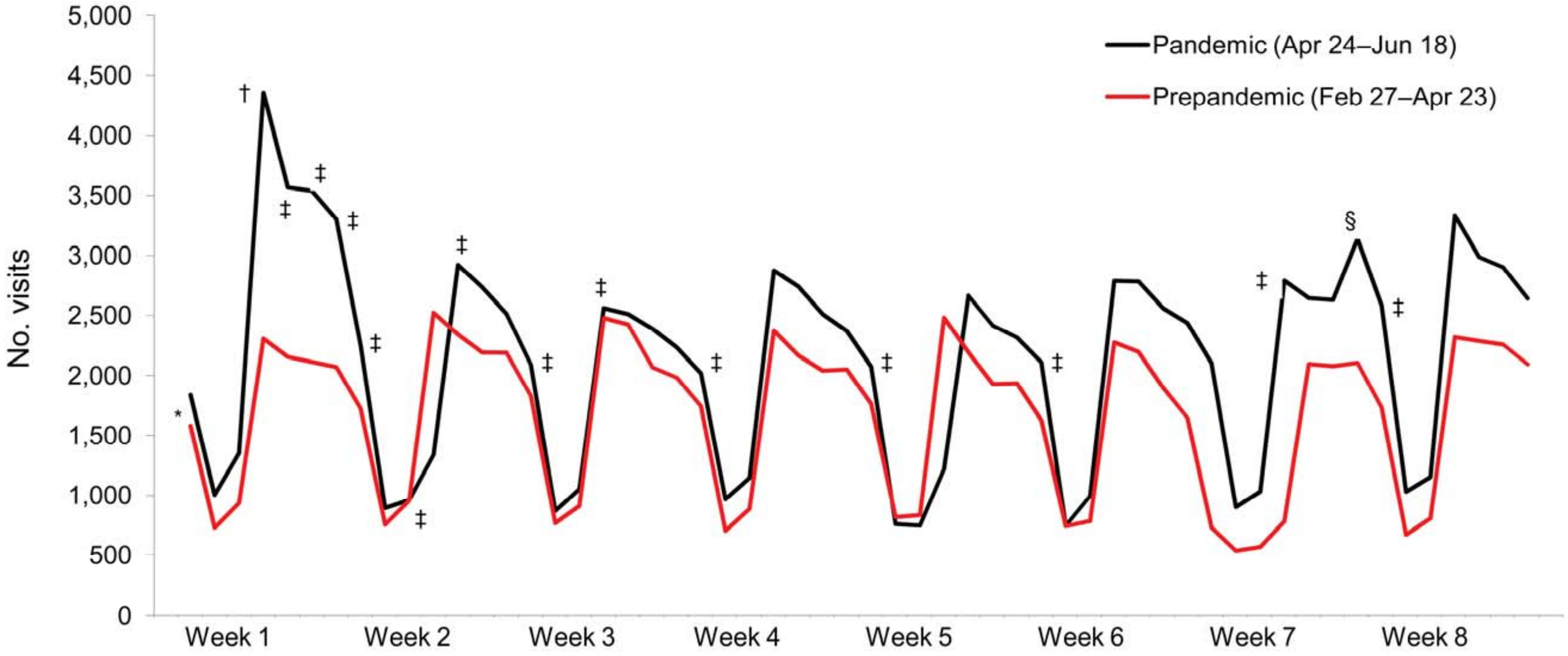

Technical Appendix Figure 1. Visits to the National Travel Health Network and Centre (NaTHNaC) website 8 weeks before and 8 weeks after recognition of pandemic (H1N1) 2009 (prepandemic and pandemic periods, respectively), United Kingdom. Noteworthy dates during the first 8 weeks of pandemic influenza are highlighted. Weeks refer to the dates during 2009 before recognition of the pandemic and weeks during it.

Prepandemic period: week 1, 27 Feb–5 Mar; week 2, 6–12 Mar; week 3, 13–19 Mar; week 4, 20–26 Mar; week 5: 27 Mar–2 Apr; week 6, 3–9 Apr; week 7, 10–16 Apr; week 8, 17–23 Apr. Pandemic period: Week 1, 24–30 Apr; week 2, 1–7 May; week 3, 8–14 May; week 4, 15–21 May; week 5, 22–28 May; week 6, 29 May–4 Jun; week 7, 5–11 Jun; week 8, 12–18 Jun. *First situation update from the World Health Organization: April 24; †first clinical update post on the NaTHNaC website: April 27; ‡clinical updates posted on the NaTHNaC website; §worldwide influenza pandemic alert level raised to phase 6 by the World Health Organization: June 11.

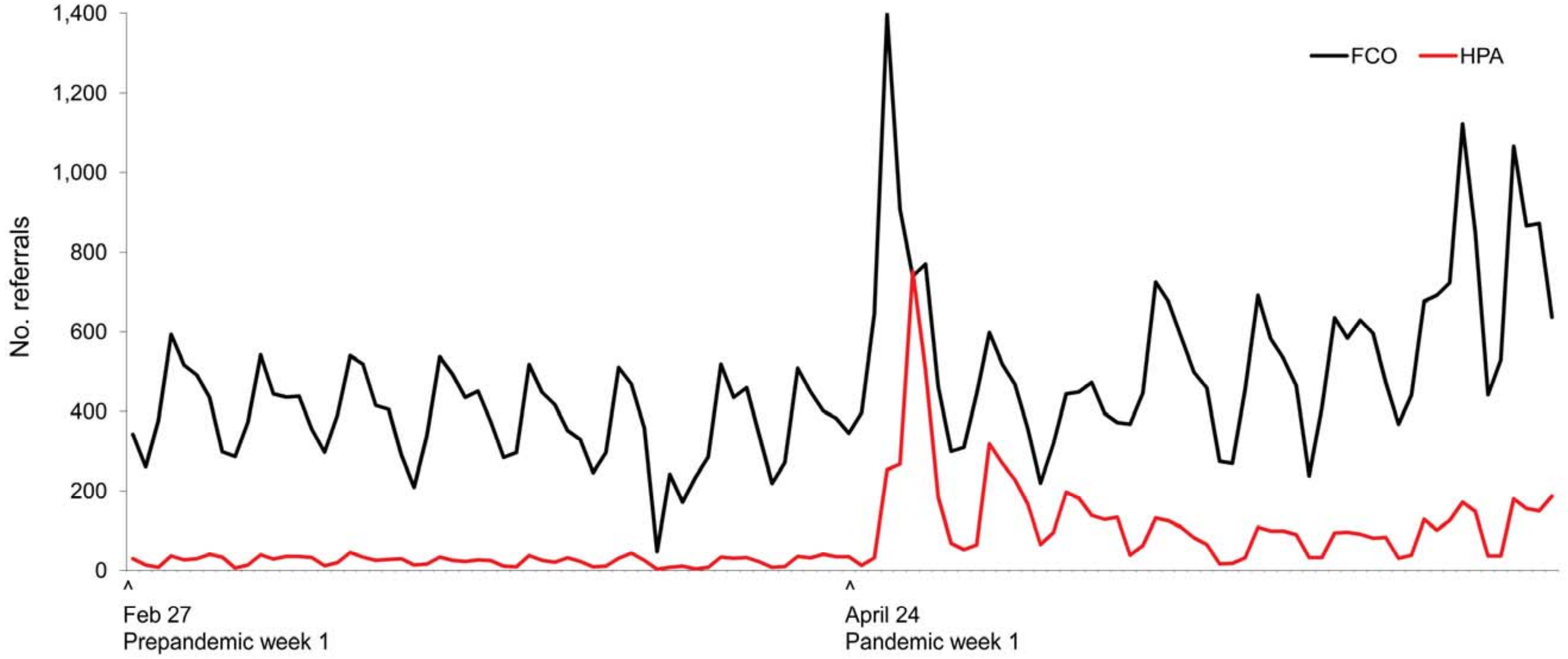

Technical Appendix Figure 2. Websites referring traffic to the National Travel Health Network and Centre website before and during recognition of pandemic (H1N1) 2009 (prepandemic and pandemic periods, respectively), United Kingdom. Prepandemic period, February 27–April 23, 2009; pandemic period, April 24–June 18, 2009; FCO, Foreign and Commonwealth Office; HPA, Health Protection Agency.
